# Magnetically recoverable bagasse-activated carbon composite anodes for sediment microbial fuel cells: enhanced performance in chromium-contaminated soil remediation

**DOI:** 10.1039/d5ra02890f

**Published:** 2025-08-26

**Authors:** Yanan Zhao, Wenlong Hui, Jian Wang

**Affiliations:** a Cangzhou Normal University Hebei 061000 China wangjian0902@163.com

## Abstract

With the increase in the use of chromium-containing products and the discharge of industrial wastes, the phenomenon of soil chromium pollution is becoming increasingly serious. Sediment microbial fuel cells (SMFCs) can be used for soil remediation In this study, the effect of SMFCs on the remediation of chromium-contaminated soil was studied. Furthermore, in order to improve the repair effect, composite electrodes were investigated as anodes to evaluate their impact on SMFC performance, with graphite felts used as a cathode. The composite electrodes were conductive bagasse-activated carbon (CBAC) composite electrode and magnetized conductive bagasse-activated carbon (MCBAC) composite electrode. An SMFC experimental group (CBAC anode denoted as SMFC-1 and MCBAC anode denoted as SMFC-2) and a control group (graphite felt anode denoted as SMFC-3) were constructed. Results showed that after activation pyrolysis, the material had an obvious pore structure, the specific surface area increased, and the surface functional groups were abundant. However, in order to improve the recovery of electrode materials, magnetization was performed on CBAC. After magnetic modification, the addition of iron oxide (γ-Fe_2_O_3_) increased the graphitization degree (Raman *I*_D_/*I*_G_ = 0.9) and reduced the charge-transfer resistance (7.68 Ω), thereby improving the electrochemical performance of MCBAC. This result was confirmed by CV and EIS results. SMFC-2 exhibited the best electrical performance, and the maximum output voltage of SMFC-2 during the period was 0.58 V, which was better than those of SMFC-1 (0.46 V) and SMFC-3 (0.35 V). Using SMFCs as the power source could effectively drive the removal of chromium from soil, and the maximum removal rate of chromium from soil was as high as 48.86% using SMFC-2. The removal rate of SMFC-1 was 43.53%, which was much higher than that of SMFC-3 (29.97%). Through the analysis of chromium morphology, it could be seen that SMFCs could effectively reduce the effective form of chromium in soil. Compared with the initial form, SMFCs effectively reduced the content of the bioactive form of chromium in soil. In summary, SMFCs can effectively drive the restoration of chromium in soil and provide an intentional reference for the restoration of heavy metals in soil.

## Abstract 2

Modified electrode prep: sodium alginate–agar mixed with 18 g CBAC/MCBAC. Graphite felt coated *via* 5 min immersion → 2% CaCl_2_ cross-linking, Core: magnetized carbon + dual cross-link → stable conductive film.

## Graphical abstract

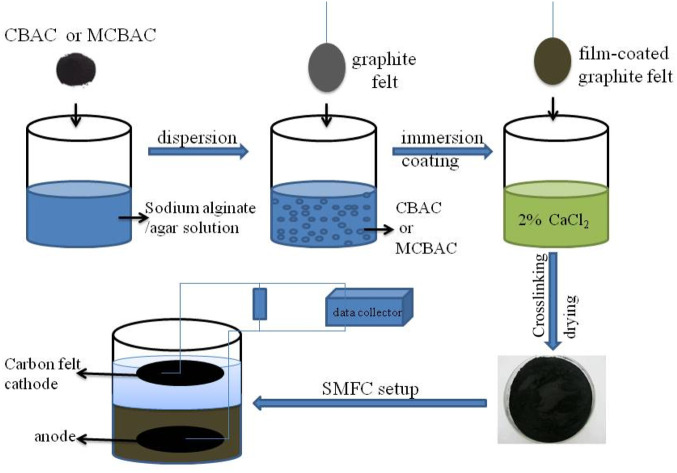

## 1. Introduction

Chromium is an important metal element widely used in industrial production, but it is also a common soil pollutant. Populations exposed to chromium pollution for a long time are more likely to suffer from tumors, and chromium entering the human body through the food chain can accumulate in the human body and cause an immeasurable impact on the human health.^1^ The remediation of chromium-contaminated soil has become one of the important issues in the field of environmental protection. Therefore, it is imperative to develop an efficient, economical and environmentally friendly remediation method for Cr-contaminated soil.^2^ Electric remediation of soil chromium pollution is a research area of great concern.^3,4^ Electric restoration technology is suitable for different types of soils, but its efficiency is affected by many factors including soil type, water content, pH value and the chemical form of chromium.^5^ The cost of electric restoration technology is relatively high, mainly encompassing power consumption, electrode material expenses and operation and management cost.^6^

The emerging sediment microbial fuel cells (SMFCs) are an efficient bioremediation electrochemical technology that can be used for soil remediation.^7^ SMFCs can utilize microbial resources in the soil and do not require any external energy input. SMFCs produce protons and electrons at the anode through microbial metabolism of organic matter, the protons are released into the anodic solution and migrate to the cathode, and the electrons arrive at the cathode through the load of an external circuit; finally, the two have an electrochemical reaction with the electron acceptor at the cathode.^8^ In theory, the SMFC anode can oxidize soluble low-priced metal ions to insoluble high-priced metal oxides, and the cathode can reduce some heavy metal ions with strong oxidation to low-priced and low-toxic substances. With the oxidation and decomposition of the substrate by electrogenic microorganisms, heavy metal ions in the soil migrate from the anode to the cathode under the action of a weak electric field, and some heavy metal ions are reduced at the cathode.^9–11^ While treating pollutants, SMFCs can also generate additional electrical energy, and through continuous in-depth research, it is found that SMFCs can remove and degrade difficult-to-degrade pollutants and toxic substances.^12–14^ Studies have shown that the SMFC technology has great potential in the remediation of Cr-contaminated soil.^15^ Researchers constructed MFCs to remediate Cr(vi)-contaminated soil and successfully achieved the reduction of Cr(vi) in the biocathode by inoculating the soil microorganisms contaminated by Cr(vi). When the initial concentration of Cr(vi) is 39.2 mg L^−1^, the reduction rate of Cr(vi) is (2.4 ± 0.2) mg (g VSS)^−1^ h^−1^.^16^ At present, there are still relatively few studies on MFCs to remediate heavy metal-contaminated soil, mainly because heavy metal ions adsorbed on the surface of soil particles are difficult to migrate, and the effect of using MFCs to reduce heavy metal ions is not ideal. However, in the process of MFCs, electricity can be generated without any external energy and secondary pollution, which has a good prospect and needs further research and exploration.

However, there are many factors affecting SMFC properties such as SMFC configuration, electrode material, electrode spacing, external part resistance, soil properties (type, concentration, moisture content, *etc.*) and temperature. However, among these factors, the electrode material has the greatest influence on the performance of SMFCs.^17–19^ It is very important to select an efficient anode electrode material. Biochar has attracted much attention in recent years due to its wide range of sources, relatively simple preparation methods and potential advantages in environmental protection and resource utilization. The preparation of activated carbon under hypoxic conditions can make it conductive. Biochar has a porous structure and a large surface area, and can be used as a soil amendment and an adsorbent. The raw materials of biochar mainly come from various biomass wastes. At present, there have been studies on the application of biochar materials in anodes to improve the performance of SMFCs.^20–23^

In this study, a composite electrode was used as the anode in SMFCs to remediate chromium-contaminated soil. A graphite felt-based composite anode material was constructed around a graphite felt with biocompatible sodium alginate and agar, while adding conductive bagasse-activated carbon (CBAC) or magnetized conductive bagasse-activated carbon (MCBAC). The anode electrode was employed for biofilm pre-cultivation, which shortened the startup time of the SMFC and enhanced the anode charge transfer capability of SMFCs. The structure and performance of the composited electrodes were investigated. The remediation effects of SMFCs with different anodes on chromium-contaminated soil were also studied. The present study will help facilitate the development of efficient and biocompatible anodes to promote the application of SMFCs in contaminated soil remediation.

## 2. Materials and methods

### 2.1 Materials

The biochar used is made of bagasse (collected in Cangzhou City, Hebei Province) as a raw material. The impurities in the bagasse were removed before carbonization, and the remaining part is crushed using a grinder and dried in an oven at 80 °C.

A graphite felt (Beijing Sanye Carbon Co., Ltd, China) was cut into a circle (*Φ* 10 cm), subsequently soaked in 1 mol L^−1^ NaOH for 24 h and then in 1 mol L^−1^ HCl for 24 h, and finally, cleaned with deionized water. Then, the graphite felt was rinsed with distilled water and dried at 50 °C for later use.

The experimental soil was collected from the surface soil of Cangzhou Normal University, and the sampling depth was 10 cm below the surface. After removing biological debris and large pieces of sand and gravel from the retrieved soil, the soil was mixed by a quarter method and stored for later use after 1 mm screening. A nutrient solution containing potassium dichromate (Cr concentration in the soil was 500 mg kg^−1^) was added to the soil that was just submerged, and the nutrient solution was stirred evenly. The physical and chemical properties of soil samples are shown in Table 1.

**Table 1:** Physical and chemical properties of the soil

Physicochemical property	Water content (%)	pH	Cr content (mg kg^−1^)	Organic content (%)	Conductivity (mS cm^−1^)
Primitive soil	3.2 ± 0.2	6.7 ± 0.2	501.23 ± 5.12	10.91 ± 1.23	0.404 ± 0.002

### 2.2 Preparation of PB and CBAC

The bagasse was loaded into a quartz tube (*Φ* 7 cm × 1.2 m), sealed and injected with nitrogen (1.5 L min^−1^), and then heated to 800 °C at 20 °C min^−1^ in a tubular furnace for 1 h. After natural cooling to room temperature in a N_2_ atmosphere, it was cleaned with distilled water to remove impurities, dried at 105 °C, ground into carbon particles of about 0.15 mm size, and stored in a dryer through a 100-mesh sieve. The pyrolyzed bagasse (PB) was obtained. Bagasse was pyrolyzed at 800 °C for 1 h under a N_2_ flow (1.5 L min^−1^), mixed with KOH (mass ratio = 1/25) and then activated at 800 °C for 1.5 h to obtain the conductive bagasse-activated carbon (CBAC).

### 2.3 Preparation of MCBAC

First, 21.02 g FeCl_3_ and 18.02 g FeSO_4_·7H_2_O (substance ratio of Fe^3+^/Fe^2+^ = 2/1) were weighed together with 15 g of CBAC and placed in a large beaker with 1200 mL of distilled water. The magnetic stirrer was used to stir at 70 °C for 1 h to make the two fully react, and then concentrated NaOH was added to adjust the pH to 10–11. Stirring was continued for 1 h, and then the beaker was covered with plastic wrap and left overnight. The supernatant was decanted, and the sediment was rinsed with deionized water to achieve a neutral pH and then dried to a constant weight to obtain magnetized conductive bagasse-activated carbon (MCBAC).

### 2.4 Electrode fabrication

**Fig. 1:**
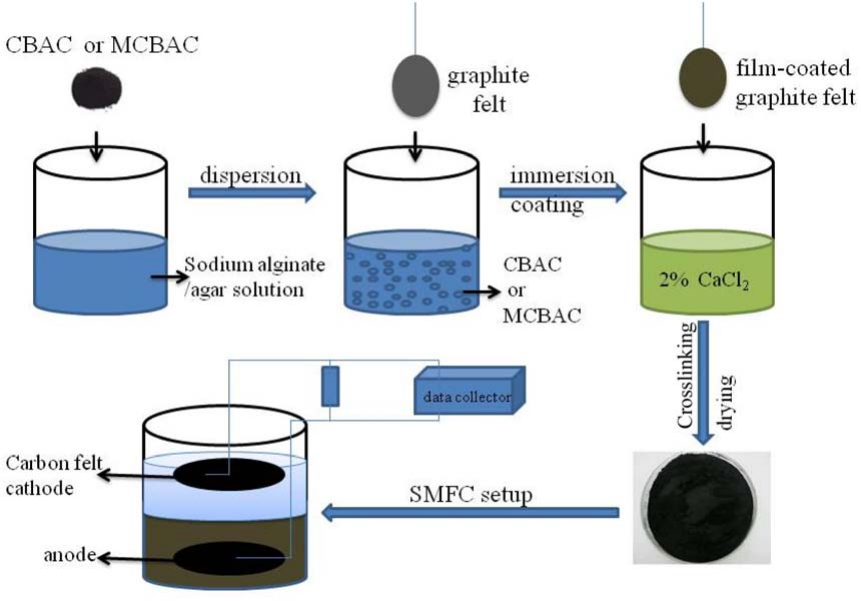
Schematic of the preparation and application of the biochar electrode composite anode.

The mixed sodium alginate–agar solution (3 g sodium alginate + 2 g agar in 100 mL deionized water) was heated to 60 °C to ensure uniformity. Then, 18 g CBAC/MCBAC was added and stirred for 30 min to form a homogeneous slurry. The graphite felt was immersed for 5 min and then cross-linked in a 2% CaCl_2_ solution for 1 h to form a stable film (thickness: ∼0.5 mm). The film-coated graphite felt was cross-linked by adding to a 2% calcium chloride solution for 1 h. After washing, the electrodes were oven-dried at 60 °C for 12 h to remove residual moisture (Fig. 1).

### 2.5 SMFC assembly and operation

Different configurations of microbial fuel cells (MFCs) have been developed based on process feasibility. Sediment MFCs (SMFCs) are particularly suitable for pollutant removal from sediments due to their configuration and extremely low cost. For this experiment, SMFCs were employed.

The anode with a connected titanium wire was placed horizontally about 2 cm way from the bottom of the sediment. The catholyte (a 50 mM K_2_HPO_4_/KH_2_PO_4_ buffer solution) was slowly added, and the cathode (carbon felt electrode) was placed on the surface of the cathode liquid to partly contact with the air phase so that the distance between the anode and cathode electrodes was 15 cm. The cathode and anode wires were connected to an external resistor with a resistance of 1000 Ω. A parallel voltage and current data collector detects the output voltage of the SMFC every 60 minutes. The CBAC composite electrode used as the anode was denoted SMFC-1, the MCBAC composite electrode used as the anode was denoted SMFC-2, and graphite felt used as the anode was denoted SMFC-3. The experiment was conducted at room temperature. Deionized water was added into SMFCs to replenish the evaporated water. Then, 10 mL nutrient solution was added to the anode every 10 days. The configuration method of nutrient solution is shown in Table 2.^24^ The organisms are enriched at the anode until the reactor outputs a stable voltage. If the output voltage of the reactor showed a downward trend, the culture solution was added in advance.

**Table 2:** Composition of the nutrient solution

Element	Concentration (g L^−1^)	Element	Concentration (g L^−1^)
Na_2_HPO_4_	4.97	MgSO_4_·7H_2_O	0.2
NaH_2_PO_4_	2.75	KCl	0.13
C_2_H_3_O_2_Na	2.0	CaCl_2_	0.015
(NH_4_)_2_SO_4_	0.56	MnSO_4_	0.028
NH_4_Cl	0.31		

### 2.6 Characterization methods of materials

A scanning electron microscope (SEM, TM3030, Hitachi, Japan) was utilized to characterize the morphology of the materials (PB, CBAC and MCBAC). The X-ray diffraction patterns of the anode samples were obtained using an X-ray diffractometer (XRD, TD3000, Tongda, China) with Cu-Kα radiation at 40 kV and 35 mA at 2*θ* values ranging from 10° to 70°. The surface functional groups of anodes were analyzed by Fourier transform infrared spectroscopy (FTIR, iS5, Thermo Fisher Scientific, China). The wave number ranges from 400 cm^−1^ to 4000 cm^−1^. Thermogravimetric analysis (TG) was performed using a thermal analyzer (TG 209F3, Netzsch, Germany) under a N_2_ atmosphere from 30 to 800 °C at a heating rate of 10 °C min^−1^. Raman spectroscopy was performed using a Raman spectrometer (QE65000-Raman, Ocean Optics, US) with a 532 nm laser. Spectral data were collected over the wavenumber range of 500–2500 cm^−1^ with a spectral resolution of 2 cm^−1^.

The electrochemical test of the materials was carried out using an electrochemical workstation (CHI660E, Shanghai Chenhua, China) in a three-electrode system. The anode, a platinum wire electrode, and a saturated calomel electrode (SCE) were used as the working, counter, and reference electrodes, respectively. The cyclic voltammetry (CV) experiments and Tafel curves were used to determine the electrochemical behaviour of the prepared electrodes. The CV was performed in a scanning range of −0.6 V to 0.8 V at a scanning speed of 0.05 V s^−1^.

To verify the reusability of the material, the MCBAC/CBAC composite electrode was placed in the treated soil. After 1 hour, it was removed with the help of neodymium magnet (surface magnetic field strength: 0.5 T). The weight of the electrode before and after the removal process was measured to calculate the recovery efficiency.

### 2.7 Performance evaluation metrics

The voltage of SMFCs was collected using a data collection system (RBH851, China), and the external resistance is 1000 Ω.

The external resistance was initially 10 000 Ω, and polarization curves were measured by varying the resistance from 10 000 Ω to 50 Ω (stabilization time: 30 min per resistance), and the voltage at both ends of the load resistance was measured after stabilizing at each resistance value. The output power density and current density were calculated using the following formula:Circuit current: *I* = *U*/*R*;Current density: *J* = *I*/*A* = *U*/(*R* × *A*);Output power: *P* = *U*^2^/*R*;Power density: *Q* = *P*/*A* = *U*^2^/(*R* × *A*).*U* is the output voltage (V), *R* is the external resistance (Ω), and *A* is the effective projected area of the anode (m^2^). The maximum output power density of SMFC was estimated by a polarization curve, and the internal resistance of SMFCs was calculated by a slope method of linear region in the polarization curve.

The CV and the electrochemical impedance spectroscopy (EIS) were performed using an electrochemical workstation in a three-electrode system, with a saturated Ag/AgCl electrode and a carbon felt cathode as the reference and counter electrodes, respectively. The working electrode was the immobilized composite anode.

### 2.8 Decontamination performance measurement

The VSS content of anode before and after operation was measured by a burning loss method. The soil samples and electrode materials were sampled to investigate chromium migration during electrokinetic restoration. The pH value and conductivity of the soil were determined using a pH meter (Shanghai Yicheng Scientific Instrument, PHSJ93F) and a conductivity meter (Shanghai Yueping Scientific Instrument, DS-11A). The total chromium content was determined using an atomic absorption spectroscope (AAS-7000, Shimadzu, Japan) after soil digestion. In order to study the concentration and morphological changes of Cr induced by SMFC, the samples of soil in the experimental and control groups were taken before and after operation. In this study, the improved BCR method was adopted to classify chromium in the soil into acid-extractable state, oxidizable state, reducible state and residual state.^25^

In order to find out the effect of SMFC on the morphological change of Cr. XRD was performed to detect the soil on the anode surface before and after operation.

## 3. Results and discussion

### 3.1 Morphological characterization of electrode materials

#### 3.1.1 SEM

**Fig. 2:**
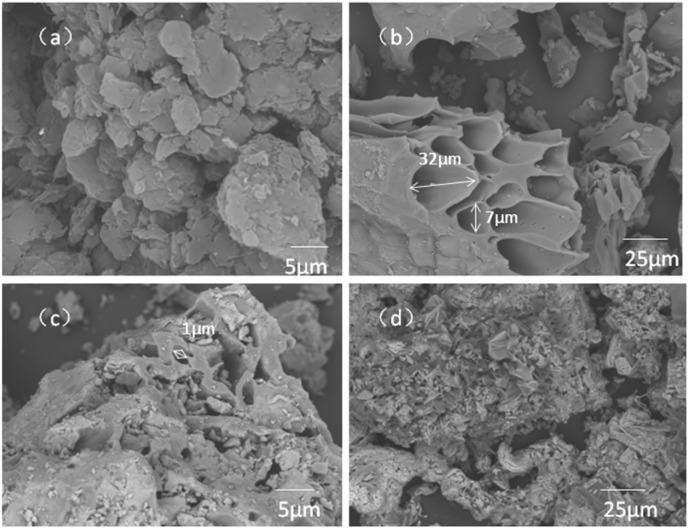
SEM micrographs of PB (a), CBAC (b), MCBAC (c) and MCBAC composite electrodes (d).

The microscopic structures of PB, CBAC, MCBAC and MCBAC composite electrodes were characterized by SEM, and the surface images are shown in Fig. 2a–d. PB (800 °C) retains a basic hierarchical framework from bagasse's vascular structure but undergoes excessive graphitization under high-temperature pyrolysis, resulting in compressed interlayer spacing. This dense, smooth structure limits the microbial adhesion sites (see Fig. 2a). The activated bagasse CBAC can be seen with a loose channel structure in the image (see Fig. 2b). KOH activation etches the carbon matrix to expand the existing pores (micropores → mesopores → macropores), forming a hierarchical porous structure rather than new layers. This enhances the surface area and connectivity, improving microbial attachment (Fig. 2b*vs.*2a). Such channels facilitate the contact between the substrate and the bacteria, and facilitate the growth of bacteria deep into CBAC. Furthermore, the rough surface is conducive to the adsorption of microorganisms, and the gully-like structure can provide shelter for microorganisms from being washed away from the surface by the medium.

In order to reduce the risk of secondary pollution caused by the loss of CBAC, the prepared CBAC was negatively magnetized, and the surface of activated carbon was loaded with nano-level ferric and trivalent ferric, in order to improve the recovery rate of activated carbon. As can be seen in Fig. 2c, the magnetic substances loaded on the surface of the activated carbon formed a coating on the activated carbon, and some of them filled into the pores, resulting in a certain extent of specific surface area loss. Magnetic modification reduces the specific surface area of activated carbon to a certain extent, and our previous studies have shown that adding a certain amount of iron is conducive to the enrichment of electrogenic microorganisms and optimization of the microbial structure on the electrode surface. Fig. 2d shows the SEM micrographs of the MCBAC composite electrode. With the cross-linked structure of sodium alginate and agar (using 2% calcium chloride solution for cross-linking), MCBAC can be well dispersed to the surface of graphite felt, and form an obvious fold structure on the surface of the composite electrode. This surface structure can effectively improve the combination of MCBAC and the substrate, so that MCBAC is not washed away from the surface by other media. Furthermore, the rough surface is conducive to the adsorption of microorganisms, and the gully-like structure can provide shelter for microorganisms.

#### 3.1.2 XRD analysis

**Fig. 3:**
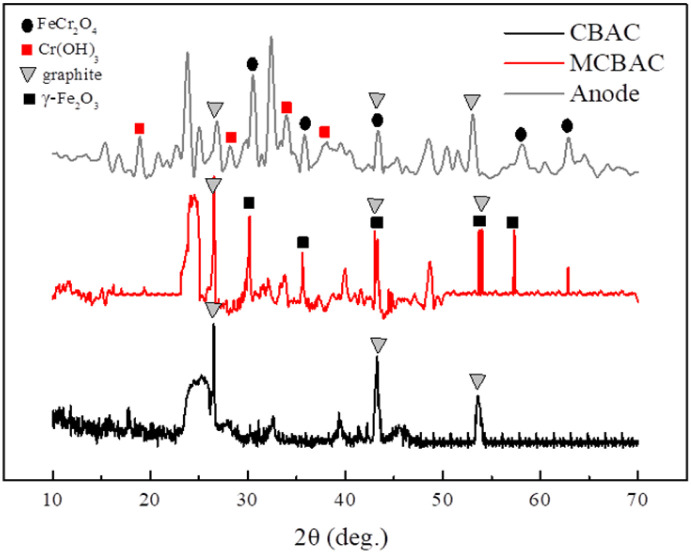
XRD pattern of the CBAC, MCBAC and MCBAC composite anodes.

The elemental composition and physical phases of CBAC, MCBAC, and MCBAC composite with the anode after remediation were analyzed by XRD. Fig. 3 shows the XRD patterns. By comparison with the PDF card, it can be seen that CBAC and MCBAC have an obvious wide peak between 20° and 30° degrees, corresponding to amorphous carbon, indicating that amorphous carbon is formed in the carbonization process of bagasse, which may affect the electrical conductivity of the material, and then can be improved by optimizing the conditions to reduce the amount of amorphous carbon. The absorption peaks at 26.5°, 42.3° and 54.7° correspond to graphite (002), (100) and (004), indicating that the bagasse has been partially graphitized after pyrolysis and has the ability to conduct electricity. From the XRD pattern of MCBAC, it can be seen that 30.1°, 35.5°, 43.1°, 53.5° and 57.0° appear on the surface after magnetization, which correspond to (220), (311), (400), (422) and (511) of γ-Fe_2_O_3_, indicating that magnetic substances such as Fe_2_O_3_ were generated on the surface of CBAC, and the magnetic modification of CBAC was successful.

The MCBAC composite electrode was removed from SMFC-2 at the end of the reaction, and the surface material of the electrode was dried for XRD analysis, as shown in Fig. 3. The results showed that there were several new peaks for the surface material. The peaks at 2*θ* = 19.0°, 27.9°, 33.6° and 40.0° are related to Cr(OH)_3_(001), (100), (101) and (012). The peaks at 2*θ* = 30.4°, 35.6°, 43.2°, 57.2° and 62.6° are related to FeCr_2_O_4_(220), (311), (400), (511) and (440), respectively.^26,27^ This indicated that Cr(vi) was reduced to Cr(iii) on the anode surface and settled on the anode surface in the form of precipitate. In addition, some Cr(vi) composite with iron on the surface to form compounds.

#### 3.1.3 TG and Raman analysis

**Fig. 4:**
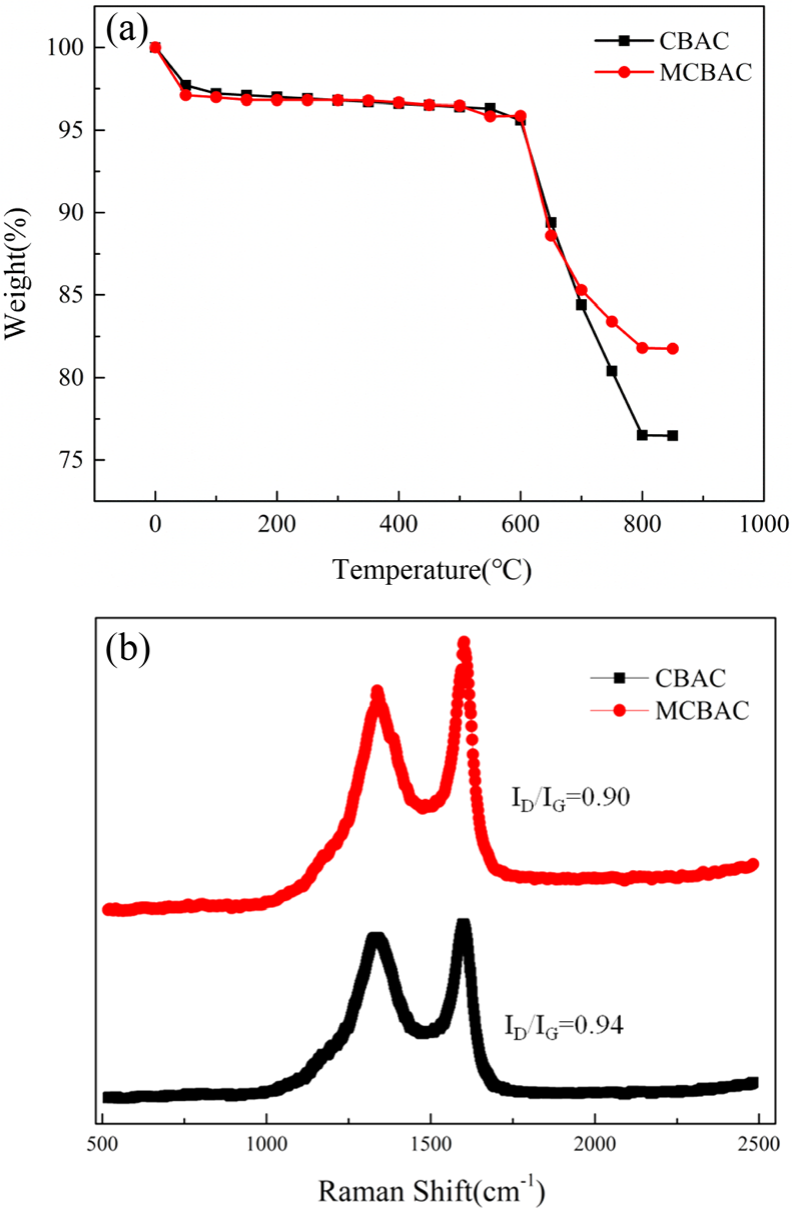
TG curve (a) and Raman curve (b) of the CBAC, MCBAC and MCBAC composite anode.

The TG curves (Fig. 4a) showed that CBAC and MCBAC have <5% weight loss below 600 °C, indicating good thermal stability. In addition, the thermal stability of MCBAC is higher than that of CBAC (weight loss at 800 °C: CBAC = 23.5% and MCBAC = 18.2%), indicating that magnetization improves thermal resistance. The Raman spectra (Fig. 4b) showed that CBAC has an *I*_D_/*I*_G_ ratio of 0.94, which decreases to 0.90 for MCBAC, indicating slightly increased graphitization and improved conductivity-consistent with a lower charge-transfer resistance in EIS (Table 3).

**Table 3:** EIS parameters fitted to the equivalent circuit model of different anodes

Anodes	*R*_1_ (Ω)	*R*_2_ (Ω)	*R*_3_ (Ω)
Graphite felt	1.40 ± 0.01	9.92 ± 0.03	45.12 ± 0.05
CBAC composite electrode	0.85 ± 0.02	8.45 ± 0.02	30.48 ± 0.07
MCBAC composite electrode	0.87 ± 0.01	7.68 ± 0.02	15.76 ± 0.03

#### 3.1.4 FTIR analysis

**Fig. 5:**
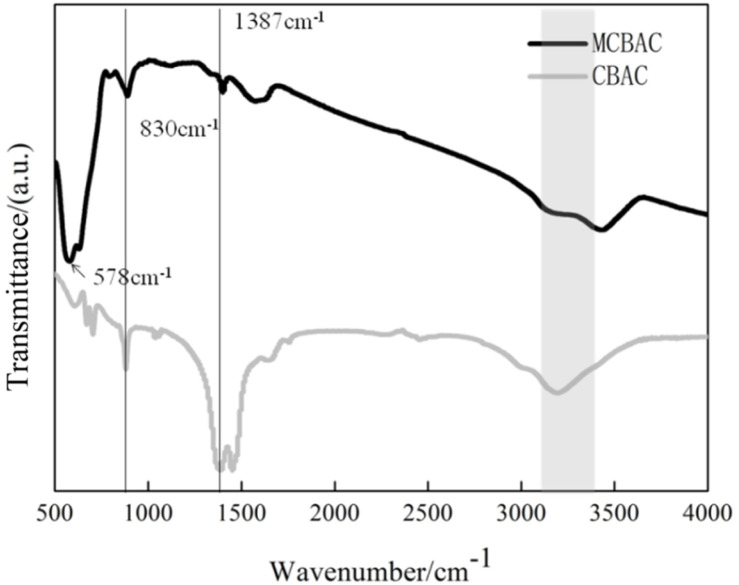
FTIR spectra of CBAC and MCBAC.

The FTIR spectra of CBAC and MCBAC are provided in Fig. 5. It could be seen from the spectrum that peaks appeared at 1387 cm^−1^ (C–H bending) and 830 cm^−1^ (aromatic C–H out-of-plane bending), confirming the presence of aliphatic and aromatic moieties. These contribute to the material's conductivity and stability. A broad peak at 3417 cm^−1^ was attributed to the O–H stretching vibrations (hydroxyl groups). These groups enhance biocompatibility by facilitating microbial adhesion and providing binding sites for chromium ions. These were all retained in both CBAC and MCBAC. A strong absorption peak of Fe–O compound appeared at 578.54 cm^−1^, indicating that there is an Fe–O bond in the magnetic activated carbon (consistent with the XRD results), which confirms the successful loading of γ-Fe_2_O_3_ during magnetization, indicating that the magnetic modification of CBAC was successful.^28^

#### 3.1.5 Recovery rate of MCBAC and CBAC

The magnetic recovery rate of MCBAC remained stable at 92% ± 3% after 5 consecutive cycles. In contrast, CBAC (non-magnetic) showed a recovery rate of only 35% ± 5% under the same conditions due to particle dispersion in soil. This high recovery efficiency addresses a key challenge in biochar-based soil remediation.

### 3.2 Bioelectrochemical characterization

#### 3.2.1 Cyclic voltammetry curve

**Fig. 6:**
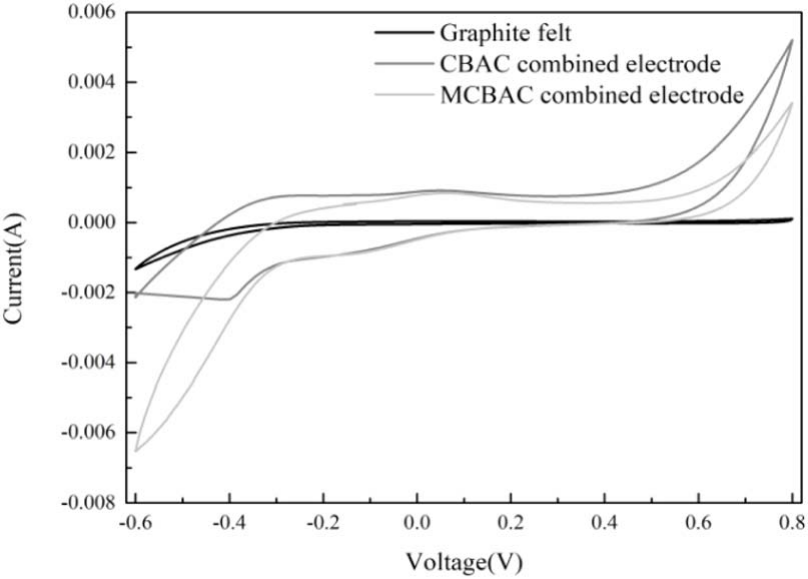
Cyclic voltammetry curves of graphite felt, CBAC composite electrode and MCBAC composite electrode anodes.

The cyclic voltammetry curves for each anode materials at a scan rate of 5 mV s^−1^ are shown in Fig. 6. The closed-loop area of the cyclic voltammetry curve of different anodes is significantly different. The MCBAC composite electrode curve has the largest closed area, while the CBAC composite electrode curve has a smaller closed area. This is caused by the negative magnetic field on the surface of CBAC. During the negative magnetic process, Fe_3_O_4_ is formed on the surface of CBAC, and the high conductivity of Fe_3_O_4_ may promote electron transfer, resulting in an increase in the current density of CV.^29,30^ The graphite felt anode has a much smaller closed area. It indicates that the composite electrode anodes possess strong electron transfer capacity and electrochemical activity. The key reason is that the surface morphology and internal structure of the electrode are obviously changed by the addition of activated carbon, which not only facilitates the microbial immobilization on the anode to form biofilm but also increases the electrochemical active sites of the electrode in contact with the microorganisms.

#### 3.2.2 Electrochemical impedance spectroscopy

**Fig. 7:**
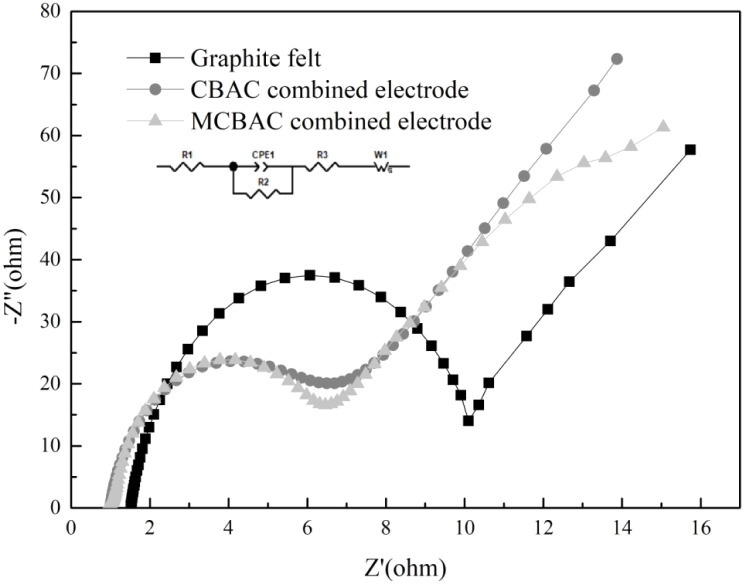
Nyquist plots of graphite felt, CBAC composite electrode and MCBAC composite electrode anodes.

EIS was performed on the anodes. The obtained Nyquist plots are shown in Fig. 7, where the inset is an equivalent circuit. *R*_1_, *R*_2_ and *R*_3_ represent the solution ohmic resistance, charge-transfer resistance and diffusion resistance, respectively. It can be seen that the solution ohmic resistance of all the SMFCs is similar, but the charge-transfer resistance and the diffusion resistance are significantly different. As the SMFC is a microbial electrochemical system, microorganisms play an important role in the SMFC. In this case, the charge-transfer resistance is particularly significant. It is related to the charge transfer efficiency between the electricity-generating bacteria and the anode, thereby affecting the electricity-generating performance of SMFCs. According to Table 3, the charge-transfer resistance of the CBAC composite electrode was 8.47 Ω, which is a bit higher than that of the MCBAC composite electrode (7.68 Ω), and the diffusion resistance of CBAC composite electrode (30.48 Ω) was higher than that of the MCBAC composite electrode (15.76 Ω). For the graphite felt anode, its charge-transfer resistance (9.92 Ω) and diffusion resistance (45.12 Ω) were 1.29 times and 2.87 times that of the MCBAC composite electrode, respectively. The charge-transfer efficiency of the electrons produced by microorganisms to the graphite felt anode was less than that of the CBAC composite electrode anode. The charge-transfer resistance was related to the formation of biofilms on the anode, the activity of microorganisms, and the way charge transfers from microorganisms to the anode. In general, the lower of the charge-transfer resistance could bring about an easier electron transfer rate, which is more conducive to the transfer of electrons generated by microorganisms to the anode surface. This change may be due to more contact sites and new binding sites from negative tape, resulting from conductive biochar modification, which is consistent with the results of other electrode modifications.^31,32^

### 3.3 Performance of SMFCs

**Fig. 8:**
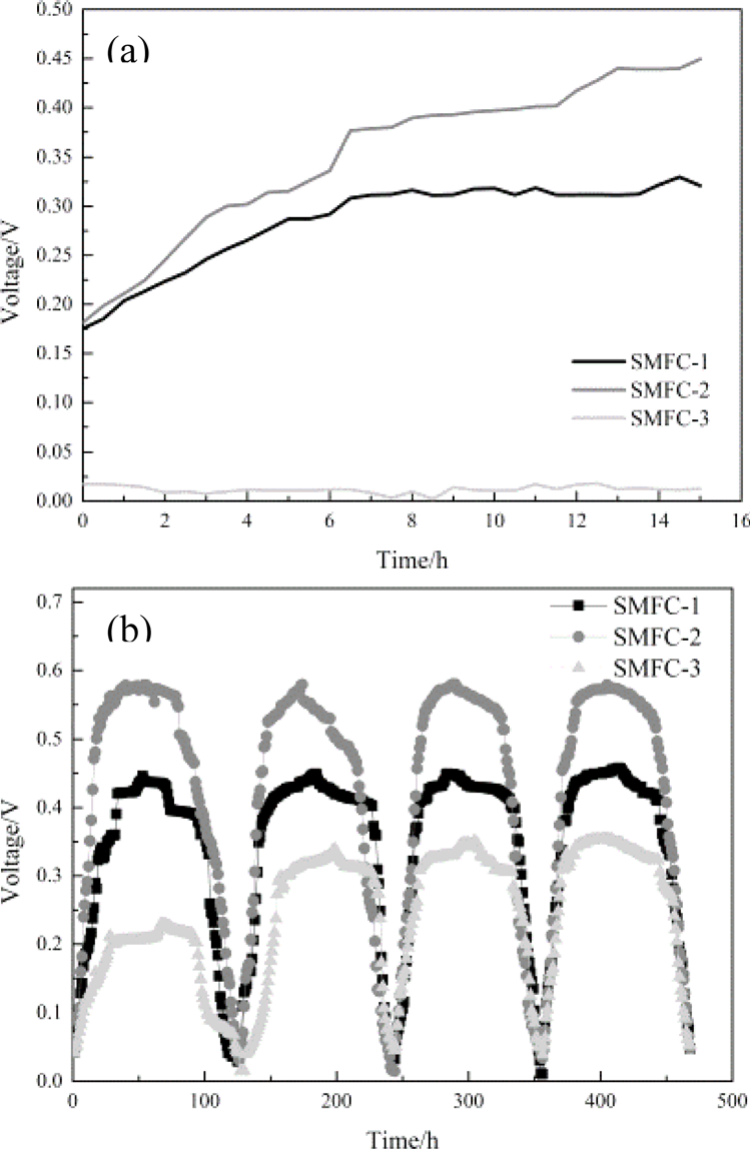
Power generation performance of SMFCs with different anodes: (a) initial voltage and (b) voltage after the fourth cycle.

SMFCs with a carbon felt cathode were cultured in batch. The start up and running process of SMFCs are shown in Fig. 8. All the anodes adsorbed and immobilized bacteria for 2 days before SMFC assembly. Fig. 8a shows that compared with SMFC-3, SMFC-1 and SMFC-2 can greatly reduce the SMFC start-up time. The SMFC with composite electrode had a shorter start-up time and higher voltage. SMFCs with composite electrode anodes had a voltage of around 180 mV at the beginning. However, the voltage of the SMFC with graphite felt anode has not increased from 30 mV and the battery has no signs of starting, until the SMFC ran to the third cycle, while SMFC-1 and SMFC-2 became stable almost in the first cycle. The start-up results reveal that successful immobilization of bacteria on the composite anodes enable the rapid formation of an anode biofilm, which does not require more time for microorganisms to adapt and grow into a biofilm after the SMFC assembly. Start-up times are greatly reduced, resulting in time and cost savings.

In the experiment, as the substrate was continuously consumed, the output voltage of SMFC dropped sharply. After the nutrient solution was injected every 10 days, the output voltage of SMFC would rise rapidly. Fig. 8b shows the voltages of SMFCs after the fourth cycle. When the SMFC operation reaches a stable level, it could be seen from Fig. 8b that SMFC-2 had the best electrical performance, the maximum output voltage of SMFC-2 during the period was 0.58 V better than 0.46 V of SMFC-1, and significantly improved compared with 0.35 V of SMFC-3.

**Fig. 9:**
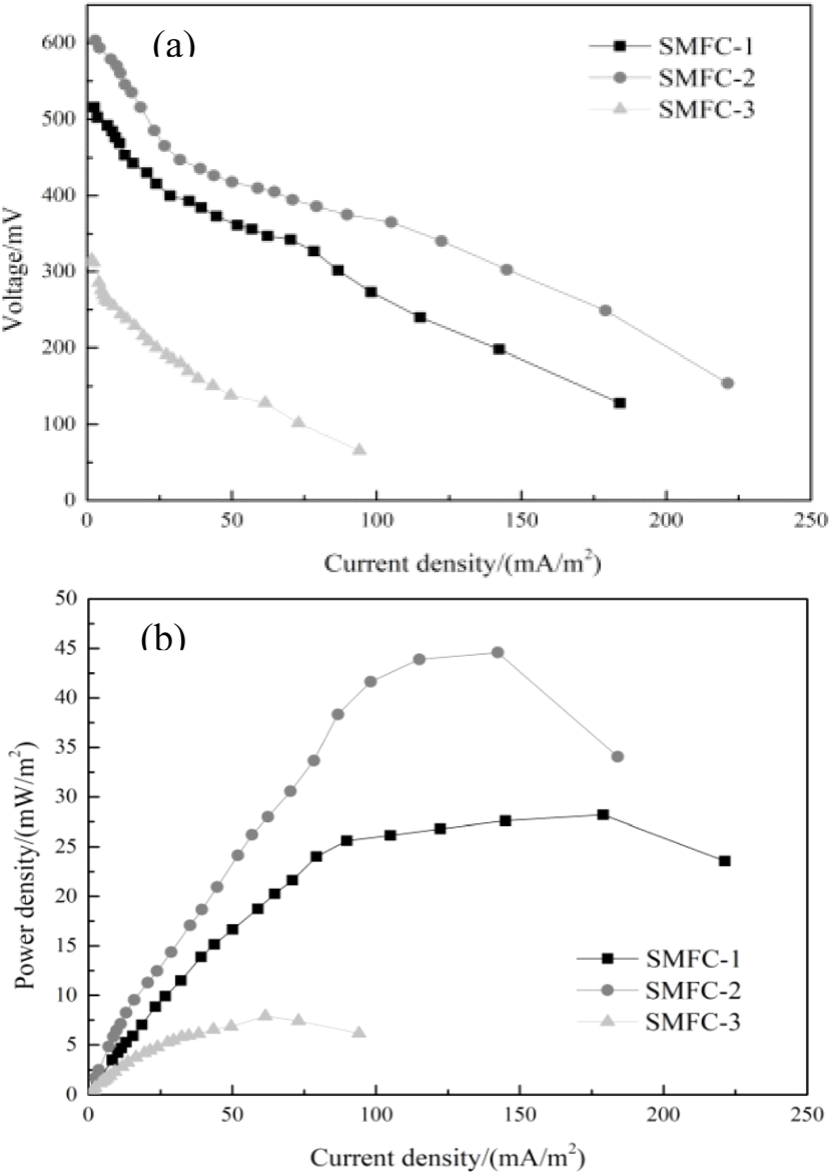
Power generation performance of SMFCs with different anodes: (a) initial voltage; (b) voltage after the fourth cycle.

The power density and polarization curves of SMFCs at the last cycle are compared in Fig. 9a and b. The maximum power density generated by SMFC-2 was greater than that of SMFC-3. The power density of SMFC-2 (44.58 mW m^−2^) is 5.65 times that of SMFC-3 and 1.58 times that of SMFC-1. The addition of CBAC has improved the power generation performance. The polarization curve was linearly fitted to obtain the internal resistance of SMFCs. The calculated internal resistances of the SMFC-1 and SMFC-2 were 215.2 Ω and 173.4 Ω, respectively, while the SMFC-3 got a calculated internal resistance of 355.3 Ω (Fig. 9b). It can be seen that the internal resistance affects the output power, and the smaller internal resistance allows SMFC to have better power generation performance. The external resistance has a great influence on the power of SMFCs. The resistance value varied with the external resistance. When the resistance value exceeded a certain critical value, the output power of SMFCs decreased with the increase in the resistance value. Therefore, too high or too low external resistance was unfavourable for SMFCs. This is because the greater the external resistance, the lower the anode potential, which can make SMFCs have a higher output voltage. However, higher external resistance would reduce the electrochemical activity of the electro-generative microorganism, so that the SMFCs could not achieve the electrical production performance. Therefore, an appropriate external resistance should be maintained to ensure that the SMFC has a better power generation.

VSS measurements showed that the anode of SMFC-2 had 54% higher biomass (12.6 μg VSS cm^−2^) than SMFC-1 (8.2 μg VSS cm^−2^), indicating better biofilm formation. This was attributed to MCBAC's hierarchical mesopores, which provide shelter and adhesion sites for microbes.

### 3.4 Effect of electric remediation of Cr-contaminated soil

#### 3.4.1 pH

**Fig. 10:**
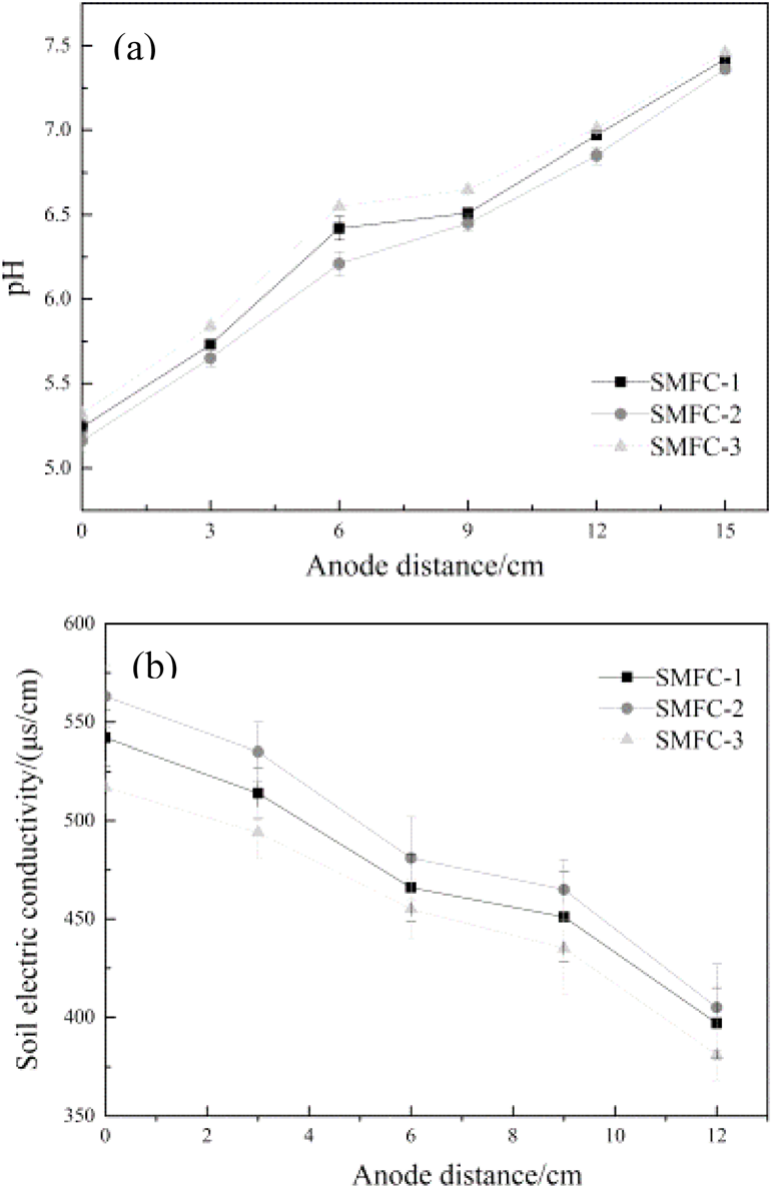
pH (a) and electrical conductivity (b) of soil in different areas.

The pH value of the original soil in all SMFCs was 6.7. However, the pH value of each zone of the anode changed after electric restoration, as shown in Fig. 10a. After repair, the environment near the anode was less acidic while the environment near the cathode was more alkaline. This is because the microorganisms in the anode oxidized to produce H^+^, which will be transferred through the soil to the cathode to combine with oxygen and be consumed, but the soil will resist the migration of H^+^, so the pH value shows a trend of gradual increase from the anode to the cathode. By comparing different groups, the SMFC-2 had the lowest pH soil environment. According to the electricity generation data (Fig. 8b), the microorganisms in SMFC-2 with higher electricity generation will produce more H^+^, resulting in a lower pH. In addition, a similar conclusion can be drawn by observing the changes in soil electrical conductivity, as shown in Fig. 10b. Because the anode produces a large amount of H^+^, the conductivity will be at a higher level, further improving the conductivity of the soil in the anode region. A lower pH could lead to an increase in the solubility of chromium, but the production of excess electrons will facilitate the change of Cr(vi) to Cr(iii), thus reducing the toxicity of chromium. With the progress of the experiment, H^+^ and dissolved chromium will migrate under the action of electric field. In the process of migration, the pH will continue to increase, and chromium will gradually precipitate, resulting in lower conductivity in the region closer to the cathode.

#### 3.4.2 Migration and removal of chromium

**Fig. 11:**
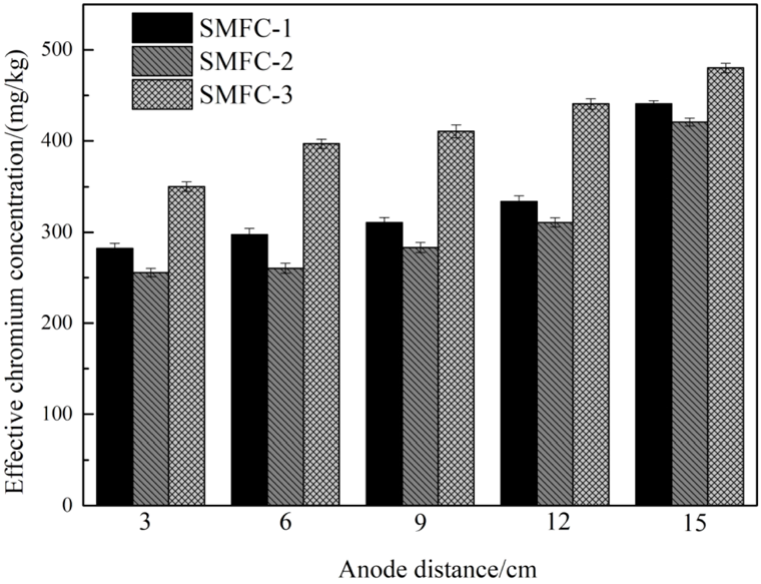
Effective concentrations of chromium in soil in different areas.

Through the use of SMFCs for electric remediation of chromium, the effective state of chromium in the soil changed significantly compared to the original soil, and the results are shown in the Fig. 11. The chromium content in the soil changed from the state of uniform in all regions at the beginning to the greater removal rate of chromium near the anode region, followed by the middle region and the lowest removal rate of chromium in the cathode region. The results showed that chromium in the soil had directional migration, which was consistent with the effect of traditional electro-kinetic remediation. This conclusion is also similar to the results of other studies on Cr removal by the MFC.^33–35^ This is due to the metabolic reaction of the anode microorganisms to produce H^+^, which is transferred to the cathode to be consumed in combination with oxygen, resulting in the dissolution of chromium in the acidic environment of the anode region. In the process of migration to the cathode under the action of the electric field generated by SMFCs, the soil pH increases and the dissolved minerals gradually precipitate and accumulate, resulting in a low removal rate of chromium in the cathode region. The results show that the weak electric field generated by SMFCs can provide power for electro-kinetic repair.

The concentration of chromium in soil in both groups decreased compared with that before operation, indicating that SMFCs had a repairing effect on chromium-contaminated soil. Before operation, the concentration of chromium in the soil was 500.00 mg kg^−1^. At the end of the experiment, the chromium removal rate in the near-anode region was the highest. The removal rates of chromium in SMFC-1, SMFC-2 and SMFC-3 were 43.53%, 48.86% and 29.97%, respectively. Other regions also showed similar trends. While the farther away from the anode zone, the removal efficiency was lower. The experimental results showed that the addition of CBAC can effectively improve the removal efficiency of chromium in soil to a certain extent. The composite material improved the electrical performance of SMFCs, and produced more H^+^ to enhance the migration and removal of chromium. However, the removal of chromium might not only be due to electric repair, and the soil was rich in a large number of microorganisms, some of which removed chromium from the environment through biochemical metabolism.

Compared with previous studies, such as Zheng *et al.*^36^ in which MFCs were used to remove Zn (with a removal rate of 25%) and Cd (with a removal rate of 18%) from paddy fields and Zhang *et al.*^37^ in which tobacco petiole pyrolytic biochar was used to remove Cr (with a removal rate of 21%), our MCBAC anode achieves better removal rate.

#### 3.4.3 Morphological distribution of chromium

In order to reduce the toxicity of chromium in addition to the total content of chromium, it is also an important purpose to reduce the toxicity of chromium in soil. In order to explore the effect of electro-kinetic restoration of SMFC on the toxicity of chromium in soil, chromium in soil is divided into four forms: acid-extractable state, oxidizable state, reducible state and residual state. Among them, the acid-extractable state refers to the fact that heavy metals combine with carbonate minerals to form co-precipitates, which are easy to transform and migrate and are the decisive factor for the mobility of heavy metals. The oxidizable state refers to the chelation of heavy metals with organic matter in the soil or their combination with sulfide minerals, which only under strongly oxidizing conditions will they decompose and be utilized by organisms. The reducible state refers to the situation where heavy metals are encapsulated by iron–manganese colloidal films or adsorbed by iron–manganese oxides and bonded by relatively stable ionic bonds. The residual state refers to the long-term stable existence of heavy metals in the lattices of silicates, primary and secondary minerals, which is stable for a long time.

**Fig. 12:**
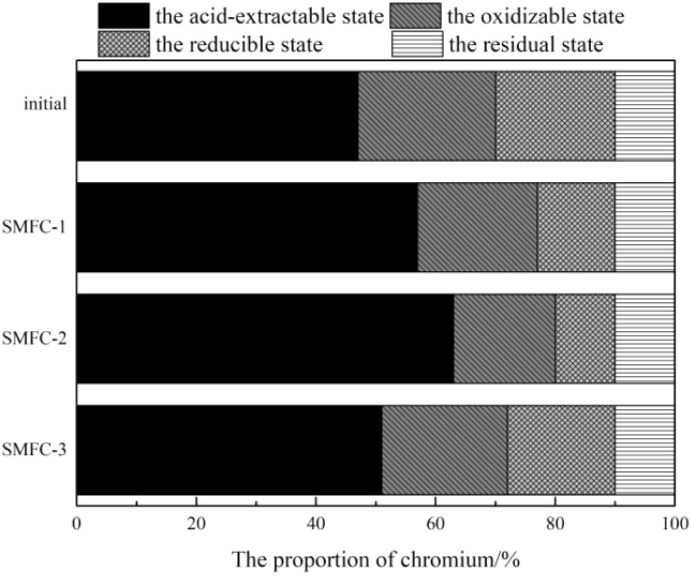
Distribution of chromium states in soil.

The morphological distribution of chromium in soil before and after restoration is shown in Fig. 12. Compared with the initial soil, the proportion of chromium in the acid-extractable state increased significantly in all experimental groups, while the proportion of oxidizable state and reducible state decreased continuously. The residual state changed little before and after the reaction. The largest proportion was the acid-extractable state and the smallest was the residual state. This was because the pH value of the soil affects the form and migration of chromium. Under low pH conditions, the reducible state was activated and transformed into the oxidizable state, while the oxidizable state was transformed into the acid-extractable state. And then, the mobile fractions of chromium migrates under the electric field force. These fractions accumulate preferentially near the cathode, thereby elevating the proportion of the migratory state (free ions) in the cathodic zone. However, inert chromium combines with the mineral lattices in the soil and is generally difficult to release, hence the changes in the residual state were not significant. This showed that SMFCs could change the form of chromium, and the chromium in the migratory state in SMFCs showed a significant increase, while the proportion of other forms keeps decreasing. SMFC-2 exhibited the optimal repair performance, as evidenced by a 16.10% increase in the acid-extractable chromium fraction. In contrast, SMFC-1 and SMFC-3 showed increases of only 10.21% and 4.10%, respectively, relative to the initial condition. The results showed that the SMFC with better electrical performance could effectively promote the morphological transformation of chromium in the soil, increase the leaching rate of chromium, and facilitate the removal of pollutants in the soil.

### 3.5 Discussion on the mechanism of chromium removal

The mechanism analyses of chromium removal in SMFCs are as follows.

#### 3.5.1 Electrochemical mechanisms: enhanced electron transfer *via* material properties

Increased graphitization: Raman spectroscopy shows that MCBAC has a lower *I*_D_/*I*_G_ ratio (0.90 *vs.* 0.94 in CBAC), indicating a more ordered carbon structure (Fig. 4b). This reduces electron transport resistance within the material, as confirmed by EIS results. Reduced charge-transfer resistance: the EIS results (Table 3) show that MCBAC has a charge-transfer resistance of 7.68 Ω, 9% lower than that of CBAC (8.45 Ω) and 23% lower than that of graphite felt (9.92 Ω). This facilitates faster electron transfer from microbial cells to the anode surface, as reflected in larger CV loop areas (Fig. 6), which correspond to a higher electrochemical activity.

Role of γ-Fe_2_O_3_: XRD confirmed γ-Fe_2_O_3_ loading in MCBAC. γ-Fe_2_O_3_ acts as an electron shuttle: Fe^3+^ is reduced to Fe^2+^ by microbial electrons (supported by a 17% increase in Fe^2+^ in MCBAC post-reaction, XPS Fe 2p spectra), and Fe^2+^ further reduces Cr(vi) to Cr(iii) (consistent with the XRD peaks of FeCr_2_O_4_, Fig. 3). This secondary electron transfer pathway complements direct microbial reduction, accelerating Cr(vi) removal.

#### 3.5.2 Biological mechanisms: microbial community and metabolic activity

The enhanced remediation efficiency is tightly linked to the enrichment of electrogenic microorganisms and their metabolic activity: VSS measurements show that MCBAC anode regions have 54% higher biomass than CBAC, indicating better metabolic activity.

#### 3.5.3 Coupled migration and transformation of chromium

The directional migration and speciation of Cr are driven by SMFC-generated electric fields and pH gradients:^24^ pH-driven transformation: lower anode pH (5.1) solubilizes Cr(vi) from reducible fractions (bound to Fe–Mn oxides), as shown by BCR analysis; reducible Cr decreases by 31% in SMFC-2, while acid-extractable Cr increases by 16.1% (Fig. 12). Migrated Cr(vi) is reduced to Cr(iii) at the cathode (supported by Cr(OH)_3_ peaks in the cathode-zone XRD) and precipitates, reducing bioavailability.

## 4. Conclusion

It was feasible to use the constructed SMFC as an electro-remediation unit to drive the treatment of chromium-contaminated soil. The SMFC can cause chromium in the soil to migrate from the anode to the cathode. By magnetic modification of CBAC, the graphitization was increased. This design (magnetized conductive bagasse-activated carbon anode) created a favorable electrochemical environment, resulting in reduced internal resistance (facilitating electron transfer) and consequently enhanced power generation performance of the SMFC. SMFC-2, leveraging this optimized anode, demonstrated superior performance in both power output (a key driver for repair) and individual repair efficacy. The maximum output voltage and power density of SMFC-2 reached 0.58 V and 44.58 mW m^−2^, respectively, which were increased by 65.71% and 5.65 times, respectively, compared with the original graphite felt electrode as the anode. Meanwhile, the chromium removal rate of SMFC-2 in the near-anode area was the highest, reaching 48.86%. In summary, the SMFC can provide an intentional reference for the restoration of heavy metals in soil.

## Conflicts of interest

There are no conflicts of interest to declare.

## Data Availability

Data are available upon request. The data that support the findings of this study are available from the corresponding author upon reasonable request. Requests for data should be addressed to zhaoyanan201607@163.com.
